# Spontaneous brain activity, graph metrics, and head motion related to prospective post-traumatic stress disorder trauma-focused therapy response

**DOI:** 10.3389/fnhum.2022.730745

**Published:** 2022-08-12

**Authors:** Remko van Lutterveld, Tim Varkevisser, Karlijn Kouwer, Sanne J. H. van Rooij, Mitzy Kennis, Martine Hueting, Simone van Montfort, Edwin van Dellen, Elbert Geuze

**Affiliations:** ^1^Brain Research and Innovation Centre, Ministry of Defence, Utrecht, Netherlands; ^2^Department of Psychiatry, University Medical Centre, Utrecht, Netherlands; ^3^Department of Psychiatry and Behavioral Sciences, Emory University School of Medicine, Atlanta, GA, United States; ^4^ARQ National Psychotrauma Centre, ARQ Centre of Expertise for the Impact of Disasters and Crises, Diemen, Netherlands; ^5^Department of Intensive Care Medicine, UMC Utrecht Brain Center, University Medical Center Utrecht, Utrecht University, Utrecht, Netherlands

**Keywords:** graph analysis, head motion, psychotherapy, PTSD, post-traumatic stress disorder, DLPFC, minimum spanning tree

## Abstract

**Introduction:**

Trauma-focused psychotherapy for post-traumatic stress disorder (PTSD) is effective in about half of all patients. Investigating biological systems related to prospective treatment response is important to gain insight in mechanisms predisposing patients for successful intervention. We studied if spontaneous brain activity, brain network characteristics and head motion during the resting state are associated with future treatment success.

**Methods:**

Functional magnetic resonance imaging scans were acquired from 46 veterans with PTSD around the start of treatment. Psychotherapy consisted of trauma-focused cognitive behavioral therapy (tf-CBT), eye movement desensitization and reprocessing (EMDR), or a combination thereof. After intervention, 24 patients were classified as treatment responders and 22 as treatment resistant. Differences between groups in spontaneous brain activity were evaluated using amplitude of low-frequency fluctuations (ALFF), while global and regional brain network characteristics were assessed using a minimum spanning tree (MST) approach. In addition, in-scanner head motion was assessed.

**Results:**

No differences in spontaneous brain activity and global network characteristics were observed between the responder and non-responder group. The right inferior parietal lobule, right putamen and left superior parietal lobule had a more central position in the network in the responder group compared to the non-responder group, while the right dorsolateral prefrontal cortex (DLPFC), right inferior frontal gyrus and left inferior temporal gyrus had a less central position. In addition, responders showed less head motion.

**Discussion:**

These results show that areas involved in executive functioning, attentional and action processes, learning, and visual-object processing, are related to prospective PTSD treatment response in veterans. In addition, these findings suggest that involuntary micromovements may be related to future treatment success.

## Introduction

Posttraumatic stress disorder (PTSD) is a psychiatric disorder that can develop after a person experiences or observes a traumatic stressor. Main features of PTSD include re-experiencing the trauma, avoidance of traumatic reminders, negative alterations in cognitions and mood, and increased arousal and reactivity (American Psychiatric Association [APA], 2013). Treatment of PTSD usually consists of trauma-focused psychotherapy (American Psychological Association [APA], 2017). However, the response rates are suboptimal, as symptoms tend to persist after treatment for 30–50% of patients (Bradley et al., 2005). To improve treatment response rates, it is important to increase our understanding of the (neuro)biological factors that may underlie differences between responders and non-responders prior to intervention.

Important models of the neurobiology of PTSD focus on the “fear circuit,” with hyper-responsive amygdalar and deficient medial prefrontal cortical and hippocampal function (Rauch et al., 2006; Shin et al., 2006; Hayes et al., 2012; Patel et al., 2012; Pitman et al., 2012). Other models have focused on large-scale brain network dysfunction, in particular the default mode network (DMN), central executive network (CEN) and salience network (SN) (e.g., Akiki et al., 2017). Interestingly, there is some evidence that trauma-focused psychotherapy for PTSD is related to changes in brain activity, specifically increased activity of the medial prefrontal cortex/rostral anterior cingulate cortex (ACC; Manthey et al., 2021). This is in line with research showing that psychotherapy tends to increase activity and recruitment of frontal areas, specifically the ACC, in other psychiatric disorders such as anxiety disorders and major depressive disorder (Quidé et al., 2012).

Several task-based functional magnetic resonance imaging (fMRI) studies have investigated predictors of trauma-focused psychotherapy treatment response in PTSD. For example, reactivity in several brain regions, including the anterior cingulate cortex (ACC), insula, amygdala, inferior parietal lobe (IPL), precuneus, and posterior cingulate cortex (PCC) has been reported to be associated with treatment response in a systematic review (Colvonen et al., 2017). An advantage of resting-state over task-based fMRI is that it avoids possible differences in task performance between groups confounding results. Resting-state brain activity can be characterized using the amplitude of low-frequency fluctuations (ALFF), which reflects the intensity of spontaneous brain activity in a specific frequency band (Yu-Feng et al., 2007). Recent findings suggest that resting-state ALFF contribute to the prediction of treatment response in a pharmacological intervention in PTSD (Yuan et al., 2018). Interestingly, pharmaceutical and psychotherapy interventions can have similar neurological effects in anxiety disorders (Linden, 2006; Barsaglini et al., 2014). To date, no studies have used pre-treatment ALFF to investigate treatment response to trauma-focused psychotherapy in PTSD.

While the fMRI studies described above provide valuable neurobiological information about the propensity for treatment response, they do not inform about functioning of the brain as a network. Recently the minimum spanning tree (MST) was introduced to neuroimaging network analysis to mitigate several methodological issues when comparing graphs between groups, such as differences in connectivity between groups and the selection of an arbitrary threshold for unweighted graph analysis (Stam et al., 2014; Tewarie et al., 2015b). Briefly, the MST represents the backbone of the functional connectivity matrix. As such, the MST can be used to assess global graph measures of network integration, i.e., how efficiently information is exchanged across the entire network, as well as regional network measures, such as the importance of each node in the global network. MST analysis has been shown to capture clinically relevant network differences (Stam et al., 2014), including populations with patterns of atypical arousal and attention such as patients with delirium and experienced meditation practitioners (Numan et al., 2017; van Lutterveld et al., 2017). No studies have yet applied this approach to assess propensity for trauma-focused psychotherapy treatment response in PTSD.

For fMRI network analyses, but essentially for all fMRI analyses, head motion is a serious confounding factor. A common approach is to compute time series of head motion metrics and subsequently regress these out of the fMRI time series. However, head motion can alternatively be considered a valid behavioral measure in itself, and recently there has been increased interest in micromovements of the head during scanning (e.g., Hodgson et al., 2017). It provides a relatively straightforward measure, for which it is not required to choose between a myriad of fMRI preprocessing choices, which can subsequently influence results (e.g., Strother, 2006; Gargouri et al., 2018). To the best of our knowledge, no studies have investigated head motion related to prospective psychotherapy treatment response.

In the present study, we aimed to address the aforementioned gaps in knowledge. To this end, war veterans with PTSD were scanned around the start of trauma-focused psychotherapy intervention. Moreover, PTSD symptomatology was assessed at that timepoint as well as after intervention to categorize patients as responders and non-responders to facilitate comparison between these groups. We expected to find (i) changes in spontaneous brain activity in responders compared to non-responders in areas most strongly discriminating prospective treatment responders and non-responders in a pharmaceutical PTSD intervention (precuneus, superior frontal gyrus, supplementary motor area, superior temporal area, frontal orbital cortex, and insula, Yuan et al., 2018), as psychotherapy can have similar effects on the brain as pharmacological interventions for anxiety disorders (Linden, 2006; Barsaglini et al., 2014), (ii) increased MST global network integration in responders, as there is suggestive evidence linking network integration to cognition, and better cognitive capabilities may lead to more effective treatment (Vourkas et al., 2014), (iii) differences in regional MST network characteristics, as a recent study found increased connectivity to be correlated with treatment response in PTSD (Zilcha-Mano et al., 2020). In addition, (iv) head motion was explored for its predictive value.

## Materials and methods

### Participants

In this study, a total number of 57 war veterans with PTSD were included. Study procedures matched those described in Van Rooij et al. (2016). In brief, a PTSD diagnosis was determined by a trained psychologist or psychiatrist in one of the four Military Mental Health outpatient clinics in the Netherlands. At the start of the study, participants were around the start of trauma-focused psychotherapy therapy. This included trauma-focused cognitive behavioral therapy (tf-CBT) or eye-movement desensitization and reprocessing (EMDR) therapy, which are recommended and suggested therapies for PTSD intervention (American Psychological Association [APA], 2017). Some participants received both tf-CBT and EMDR therapy sessions (see Table 1). Trained researchers applied the Clinician-Administered PTSD Scale (CAPS; Blake et al., 1995) to examine severity of PTSD symptoms at this timepoint and after 6–8 months. During this interval, patients received trauma-focused therapy. In addition, a structured clinical interview for DSM-IV axis I disorders (SCID-I/P; First et al., 1995) was conducted at these timepoints to determine any comorbid psychiatric disorders. Baseline fMRI scans and clinical interviews were performed as close as possible to the start of treatment, while the clinical interviews were repeated 6–8 months later to assess changes in symptomatology. Inclusion criteria consisted of deployment to a warzone and an age between 18 and 60 years old. Participants with a history of neurological disorders were excluded. Comorbid psychiatric disorders such as mood disorders, psychotic disorders, substance-related disorders, or any other psychiatric disorder were not considered exclusion criteria. The PTSD patients were grouped into responders and non-responders based on their reduction in CAPS scores at the second timepoint, similar to previous studies (e.g., Rubin et al., 2016; Van Rooij et al., 2016; Yuan et al., 2018; Zhutovsky et al., 2019). A participant was considered responsive if total CAPS score was reduced by at least 30 percent after intervention (Brady et al., 2000).

**TABLE 1:** Demographics and clinical data.

	Responders (*n* = 24)	Non-responders (*n* = 22)	Test-value (df)	*P*-value
Age (median, IQR [years])	36 (13)	36.5 (15)	*U* = 202	*P* = 0.172^a^
Gender (m/f)	24/0	22/0		
Handedness (left/right/ambidextrous)	1/21/2	3/17/2	1.390	*P* = 0.632^b^
*Education (0/1/2/3/6 [ISCED])*				
Own	0/0/3/16/5	0/0/7/14/1	4.078	*P* = 0.139^b^
Mother	1/0/15/4/3	1/2/10/4/3	2.805	*P* = 0.678^b^
Father	0/1/10/3/8	1/1/6/6/7	3.186	*P* = 0.583^b^
Time since last deployment (median, IQR [months])	44.5 (160)	67 (149)	*U* = 237	*P* = 0.733^a^
Number of times deployed (median, IQR)	2 (4)	2 (2)	*U* = 212.5	*P* = 0.346^a^
Early traumatic experiences (median, IQR [ETI total])	3 (4.25)	4 (7.75)	*U* = 211	*P* = 0.820^a^
*Therapy received*				
EMDR/tfCBT/EMDR and tfCBT	18/4/2	11/4/7	χ^2^(2) = 4.296	*P* = 0.117^b^
Total number of therapy sessions (median, IQR)	6.5(8.0)	8.5 (6.0)	*U* = 152.5	*P* = 0.214^a^
*Therapy received prior to scanning and CAPS*				
No therapy/EMDR/tfCBT/EMDR and tfCBT	17/5/2/0	13/3/4/1	χ^2^ = 2.442	*P* = 0.528^b^
Total number of therapy sessions (median, IQR)	0.0(1.0)	0.0(5.5)	*U* = 209.000	*P* = 0.243
**Baseline clinical scores**				
*Clinical scores at baseline (mean, SD [CAPS])*				
Total	70.33 (15.3)	70.55 (11.15)	*t*(44) = –0.053	*P* = 0.958^c^
Re-experiencing	23.21 (4.76)	22.73 (5.88)	*t*(44) = 0.306	*P* = 0.761^c^
Avoiding	22.96 (11.63)	23.23 (6.48)	*t*(36.6) = –0.098	*P* = 0.923^d^
Hyperarousal	24.17 (5.04)	24.59 (4.34)	*t*(44) = –0.305	*P* = 0.762^c^
*Comorbid disorder baseline (no. [SCID])*				
Mood disorders	12	13	χ^2^(1) = 0.382	*P* = 0.568^e^
Schizophrenia and other psychotic disorders	0	1	§	*P* = 0.478^b^
Substance-related disorders	1	1	§	*P* = 1.000^b^
Anxiety disorders	7	11	χ^2^(1) = 2.092	*P* = 0.227^e^
Somatoform disorders	0	1	§	*P* = 0.478^b^
*Baseline medication (no.)*				
SSRI	4	8	χ^2^(1) = 2.31	*P* = 0.183^e^
Benzodiazepines	7	3	§	*P* = 0.289^b^
SARI	2	0	§	*P* = 0.490^b^
Antipsychotics	2	0	§	*P* = 0.490^b^
β-blockers	0	2	§	*P* = 0.223^b^
Nicotine agonists	1	0	§	*P* = 1.000^b^
Ritalin	0	0		
**Post treatment clinical scores**				
*Clinical scores post-treatment* *(median, IQR [CAPS])*				
Total	32 (27)	67.5 (22)	*U* = 14.5	*P* < 0.001^a^*
Re-experiencing	6 (12.25)	23.5 (4)	*U* = 40.5	*P* < 0.001^a^*
Avoiding	6 (10.75)	19.5 (15.25)	*U* = 46.5	*P* < 0.001^a^*
Hyperarousal	13 (12.25)	23.5 (10.25)	*U* = 60	*P* < 0.001^a^*
*Comorbid disorder post-treatment (no. [SCID])*				
Mood disorders	4	10	χ^2^(1) = 5.007	*P* = 0.051^e^
Schizophrenia and other psychotic disorders	0	2	§	*P* = 0.212^b^
Substance-related disorders	0	2	§	*P* = 0.212^b^
Anxiety disorders	3	10	χ^2^(1) = 6.724	*P* = 0.019^e^*
Somatoform disorders	0	2	§	*P* = 0.212^b^
*Post-treatment medication (no.)*				
SSRI	5	11	χ^2^(1) = 3.919	*P* = 0.065^e^
Benzodiazepines	6	2	§	*P* = 0.243^b^
SARI	2	0	§	*P* = 0.489^b^
Antipsychotics	2	2	§	*P* = 1.000^b^
β-blockers	0	0		
Nicotine agonists	0	0		
Ritalin	0	0		

SD, standard deviation; IQR, interquartile range; ISCED, international scale for education; CAPS, clinician administered PTSD scale; SCID, structured clinical interview for DSM IV Axis II disorders; SSRI, serotonin reuptake inhibitor; SARI, Serotonin antagonist and reuptake inhibitors; EMDR, eye movement desensitization and reprocessing; tf-CBT, trauma-focused cognitive behavioral therapy.

*P < 0.05.

§: No test-value is provided by SPSS for 2 × 2 Fisher’s exact tests.

Number of cases with missing data: education mother responders *n* = 1; education mother non-responders *n* = 2; education father responders *n* = 2; education father non-responders *n* = 1; time since last deployment non-responders *n* = 1; number of times deployed non-responders *n* = 1; ETI responders *n* = 2; ETI non-responders *n* = 2; total number of therapy sessions responders *n* = 6; therapy received prior to data acquisition and number of therapy sessions prior to data acquisition non-responders *n* = 1; SCID post-treatment non-responders *n* = 1; post-treatment medication responders *n* = 1.

^a^Mann–Whitney *U* test.

^b^Fisher’s exact test.

^c^Student’s *t*-test.

^d^Welch’s *t*-test.

^e^χ2-test.

From the initial sample size of 57 PTSD patients, four were lost to follow up, two did not receive treatment and one was excluded due to her being the only female in the sample. A final participant was excluded because a resting state scan was not performed. At the analysis stage, three subjects were excluded (see section “Participants”) and the resulting sample therefore consisted of 46 PTSD patients. The study was approved by the institutional review board of the University Medical Center of Utrecht and all participants gave their written informed consent before participation in the study.

### Image acquisition

Imaging was performed on a Philips Achieva 3 Tesla Clinical MRI scanner (Philips Medical System, Best, Netherlands). A high-resolution T1 weighted anatomical scan was acquired to improve localization of the functional data with the following settings: repetition time (TR): 10 ms, echo time (TE): 4.6 ms, flip angle (FA): 8°, 200 sagittal slices, field of view (FOV) 240 × 240 × 160, matrix 304 × 299. Hereafter, 320 blood-oxygenation level-dependent (BOLD) resting-state fMRI images were acquired per subject with the following settings: TR: 1600 ms, TE: 23 ms, FA: 72.5°, FOV 256 × 208 × 120), 30 transverse slices, matrix 64 × 51, voxel size 4 × 4 × 3.60 mm, 0.4 mm gap, total scan time 8 min and 44.8 s. Participants were asked to focus on a fixation cross, let their mind wander and relax during resting-state scanning, and were provided with thorough instructions to prevent head motion during scanning.

### Data analysis

#### Preprocessing

Functional MRI data were preprocessed using the Data Processing Assistant for Resting-State fMRI (DPARSF) advanced edition (version 4.5) as part of the Data Processing and Analysis for Brain Imaging (DPABI; Yan et al., 2016) toolbox, running in MATLAB (MATLAB, 2019). The first 10 volumes were discarded for steady-state magnetization, leaving 310 volumes for further analysis. The remaining volumes underwent realignment, skull stripping using the Brain Extraction Tool (BET; Smith, 2002), co-registration to the individual structural scan, segmentation into grey matter (GM), white matter (WM) and cerebrospinal fluid (CSF) and linear trends removal. Nuisance covariate regression was performed using a 36-parameter model and included temporal censoring and global signal regression as recommended (Ciric et al., 2017). Temporal censoring was performed through spike regression based on framewise displacement (FD) limits of 0.2 mm calculated according to Jenkinson’s algorithm (Jenkinson et al., 2002). Participants with < 4 min of data after spike regression were excluded from analysis for ALFF, global and regional network analysis (two for the responder group and one for the non-responder group, Satterthwaite et al., 2013). The 36-parameter model included Friston’s 24-parameter model, i.e., 6 head motion parameters, 6 head motion parameters from the previous time point, and the 12 corresponding squared items (Friston et al., 1996), as well as the GM, WM and CSF time-courses, their derivatives, the squares of the time-courses, and the squares of the derivates. Hereafter, images were spatially normalized to Montreal Neurological Institute (MNI) space using Diffeomorphic Anatomical Registration Through Exponentiated Lie Algebra (DARTEL; Ashburner, 2007).

#### Amplitude of low frequency fluctuations

Preprocessed images were analyzed in DPARSF by calculating the amplitude of low frequency fluctuations (ALFF; Yu-Feng et al., 2007). ALFF reflects the intensity of spontaneous brain activity in a specific frequency band. ALFF was calculated for each voxel by performing a fast Fourier transform (FFT) on its time-series, square-rooting the power spectrum and averaging in the 0.01 – 0.08 Hz frequency band. ALFF values were normalized to z-scores and smoothed with an 8 mm full width at half maximum (FWHM) Gaussian kernel.

#### Global network analysis

**FIGURE 1:**
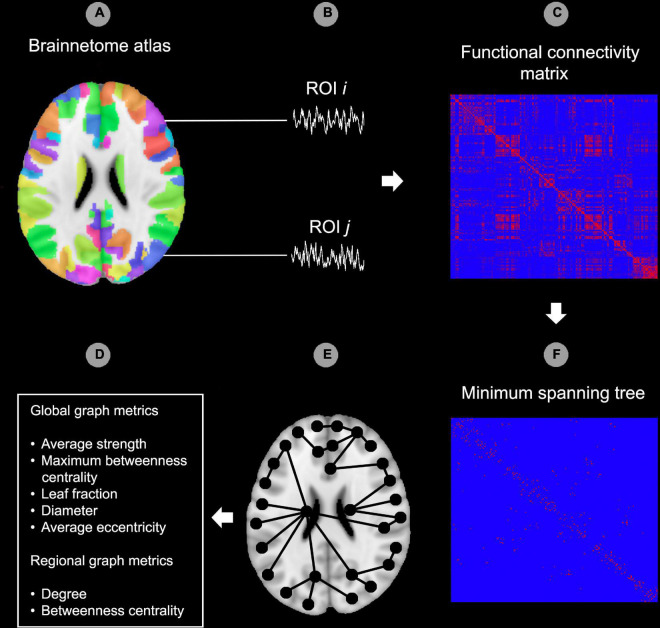
Graph analysis pipeline. In the first step, the Brainnetome atlas was applied to the functional data **(A)**. The second step consisted of extracting the time-courses for each region of interest (ROI) of the atlas **(B)**. After this, functional connectedness was calculated between all possible pairs of ROIs *i* and *j* in each subject **(C)**. Hereafter, the minimum spanning tree (MST) was constructed from the functional connectivity matrix by including the strongest connections while avoiding loops. All edges in the MST were set to 1 while edges outside the MST were set to 0 **(D)**. Image **(E)** provides a theoretical example of an MST superimposed on a template brain. As a final step, the MST network was characterized using global and regional (node specific) graph metrics **(F)**. ROI, region of interest. The reader is referred to the web version of this paper for the color representation of this figure.

**FIGURE 2:**
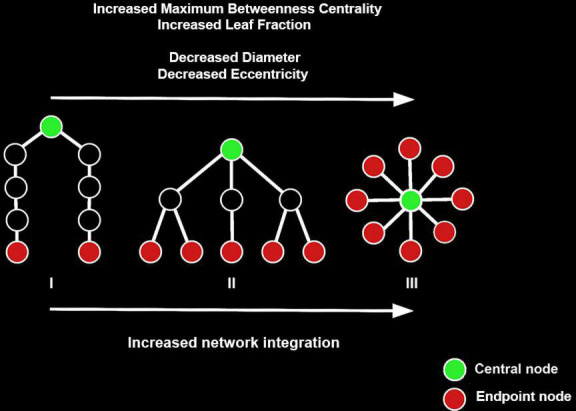
Examples of three minimum spanning trees (MSTs) consisting of nine nodes. MST structures can be found on a continuum between a path-like tree to a star-like tree, with the figure showing a path-like (I), hierarchical (II), and star-like tree (III). Path-like trees have the drawback that information does not flow easily from node to node. Star-like trees have the advantage that information can flow easily across the network and the downside that the central node might suffer from information overload. As such, a hierarchical tree is the hypothesized optimal topology. Figure adapted from van Lutterveld et al. (2017). The reader is referred to the web version of this paper for the color representation of this figure.

Preprocessed images were further processed in DPARSF by smoothing the images with an 8 mm full width at half maximum (FWHM) Gaussian kernel. Hereafter, data were filtered between 0.01 and 0.08 Hz and a functional connectivity matrix was created by calculating Pearson’s correlation coefficients between the regions of interest (ROIs) of the Brainnetome atlas (Fan et al., 2016). Eight occipital ROIs (out of a total of 246, 3.3%) were excluded from analysis as these were not included in the FOV for all participants (bilateral caudal lingual gyrus, left caudal cuneus gyrus, left middle occipital gyrus, bilateral occipital polar cortex, bilateral inferior occipital gyrus). Network topology of the functional connectivity matrix was evaluated using BrainWave software (Stam, 2018). As a first step, an MST was created for each functional connectivity matrix using Kruskal’s algorithm (Kruskal, 1956; Stam et al., 2014). The MST approach to characterize functional connectivity matrices has been utilized with various imaging modalities, including EEG (e.g., Boersma et al., 2013; Stam et al., 2014; Vourkas et al., 2014; Fraga Gonzalez et al., 2016; van Lutterveld et al., 2017), MEG (van Dellen et al., 2014; López et al., 2017) and fMRI (e.g., Ciftci, 2011; Tewarie et al., 2015a; Guo et al., 2017, 2018; Wang et al., 2018). Specifically, the MST is a binary subgraph of the functional connectivity matrix that connects all nodes (i.e., Brainnetome ROIs), such that the strongest connections in the original network are included while avoiding loops. As such, the MST is an unweighted backbone graph of the original functional connectivity matrix that is considered to reflect the functional core of the network (Stam et al., 2014). Importantly, the MST is considered to avoid several methodological issues that can arise when comparing graphs between groups, such as differences in average connectivity strength between groups for weighted graph analysis and the selection of an arbitrary threshold for unweighted graph analysis (van Wijk et al., 2010; Tewarie et al., 2015b). Negative correlations were set to 0 when calculating the MST, thus avoiding the problematic interpretation of negative blood-oxygen-level-dependent (BOLD) correlations (Stam et al., 2014; Tewarie et al., 2015b). The MST network was characterized using average connectivity strength and four graph measures of network integration (maximum betweenness centrality, leaf fraction, diameter and average eccentricity; see Table 2 for an explanation). Graph metrics except average strength were normalized for network size, yielding values between 0 and 1. Figure 1 provides a graphical representation of the graph analysis pipeline, while Figure 2 shows additional information about the theoretical range of MSTs and its associations with each graph measure.

**TABLE 2:** Definitions and interpretations of Minimum Spanning Tree (MST) graph measures (Tewarie et al., 2015b).

Minimum Spanning Tree (MST) graph measure	Definition	Explanation
Average connectivity strength	The mean of the edge weights in the original connectivity matrix that are included in the MST.	Reflects weighted connectivity strength.
Maximum betweenness centrality	The fraction of all shortest paths passing through a node. Maximum betweenness centrality indicates the highest value of betweenness centrality across all nodes in the MST.	Reflects the strength of the most important node in the MST, i.e., how crucial this node is as a hub for information flow.
Leaf Fraction	The number of nodes with one edge (i.e., ‘end-points’ in the graph) divided by the maximum possible number of nodes with one edge (i.e., the number of nodes minus 1, this indicates a starshaped graph).	Reflects to what extent the MST has a central organization. A high leaf fraction indicates that the information flow is largely dependent on hub nodes.
Diameter	The number of edges of the longest path in the MST.	Reflects the efficiency of global network organization. In a network with a low diameter, information flows efficiently between remote brain regions.
Average Eccentricity	Eccentricity of a node is defined as the maximum number of edges between that node and any other node. Average eccentricity indicates the average across all nodes.	Reflects the tendency of nodes in the network to be isolated and poorly integrated.

#### Regional network analysis

To localize potential regional differences in network organization, centrality (i.e., relative importance) of each node in the MST was assessed using degree and betweenness centrality.

Degree refers to the number of connections between a node and other nodes in the network, while betweenness centrality refers to the numbers of shortest paths that pass through a node.

#### Head motion

To investigate whether head motion is associated with response to trauma-focused psychotherapy, four resting-state motion metrics were assessed based on Jenkinson’s algorithm using output from the fMRI preprocessing pipeline (Jenkinson et al., 2002): (1) Median FD, (2) maximum FD, (3) number of motion outliers [defined as the number of timepoints for which FD > 0.2 mm), which is a commonly used threshold to identify motion outliers (e.g., Chen et al., 2018), and (4) interquartile range (IQR) of FD. As head motion has previously been reported to be associated with education level, cigarette use at the day of scanning, cigarette use the week prior to scanning, dexterity as well as age, associations of motion metrics with these variables were explored for head motion metrics that differed significantly between groups (Pardoe et al., 2016; Siegel et al., 2017). In addition, associations between head motion and anxiety (as measured with the Mood and Anxiety Symptom Questionnaire (MASQ) anxious arousal subscale) and depression (as measured with the MASQ anhedonic depression subscale) were assessed (Watson et al., 1995).

#### Sensitivity analyses residual motion and graph analysis

As fMRI-based functional connectivity and graph metrics are particularly vulnerable to residual head motion confounds (e.g., Ciric et al., 2017), several control analyses were performed: (1) for the functional connectivity matrix, the percentage of edges significantly correlating with average FD was calculated, (2) for the functional connectivity matrix, the distribution of connectivity strength – average FD correlation values was calculated, (3) associations between average FD and each of the global graph outcome metrics were assessed, (4) associations between average FD the regional graph outcome metrics were assessed for each node.

#### Data availability

Anonymized head motion and preprocessed fMRI data are available upon reasonable request from any qualified investigator.

### Statistical analysis

#### Amplitude of low-frequency fluctuations

In line with recent recommendations (Eklund et al., 2016), statistical testing was performed using a cluster-based non-parametric permutation test (SnPM13; Singh et al., 2003; Holmes et al., 2018) with 10,000 permutations. The responder and non-responder groups were tested for differences in ALFF using SnPM’s two-sample *t*-test. The cluster defining threshold was set to *P* < 0.001 uncorrected and the family wise error (FWE) correction threshold to account for multiple comparisons was set at *P* < 0.05. Covariates of no interest included age, education and mean FD (Li et al., 2014, 2019; Guo et al., 2017). Analysis was restricted to a small-volume corrected mask consisting of 5 mm radius spheres centered on the peak coordinates most strongly discriminating prospective responders and non-responders in a pharmacological intervention in PTSD [peak weight vector score ≥ 2.00 or ≤–2.00; precuneus (MNI coordinates 3, –48, 69)], superior frontal gyrus/supplementary motor area (12, –3, 75), superior temporal area/frontal orbital cortex (36, 21, –21), superior temporal area/insula (–45, 9, –15) and right superior temporal area (60, –21, 15) (Yuan et al., 2018), as psychotherapy can have similar effects on the brain as pharmacological interventions for anxiety disorders (Linden, 2006; Barsaglini et al., 2014). Whole-brain effects were also explored using the Brainnetome gray matter mask (Fan et al., 2016), with part of the posterior occipital cortex excluded from analysis as it was not included in the FOV for all participants, and for this reason 11.7% of all voxels were excluded.

#### Global network analysis, regional network analysis and head motion

Statistical testing was performed through permutation testing (Lawrence, 2015) in R version 3.6.1 and RStudio version 1.2.5042 for the global and regional network analyses as well as the head motion metrics. Outliers were identified based on exceeding 3 * IQR limits, and subsequently removed from analysis. Each analysis was corrected for multiple comparisons using false discovery rate (FDR; Benjamini and Hochberg, 1995). Supplementary Materials and Methods S1 provides detailed information. As with the ALFF analysis, part of the occipital cortex was excluded from analysis as it was not included in the FOV for all participants, and for this reason 3.3% of all nodes were excluded.

#### Sensitivity analyses residual motion and network analysis

Several sensitivity analyses were performed to assess the potential effect of confounding residual head motion on the network analyses. Potential associations between motion and the underlying functional connectivity matrix were assessed by (1) correlating average FD with connectivity strength of each edge in the functional connectivity matrix using Kendall’s tau procedure. Statistical significance was set at *P* < 0.05 and the FDR was used to account for multiple comparisons (Benjamini and Hochberg, 1995, identical to Ciric et al., 2017). The number of significant correlations was then summed and divided by the total number of edges. This established the percentage of edges significantly correlating with average FD. (2) The distribution of correlation values between connectivity strength and average FD was plotted for all edges. A distribution center close to zero indicates relatively good performance of the motion correction pipeline. (3) Correlating average FD with each global graph measure using Kendall’s tau procedure to identify potential associations between motion and the global network outcome measures. (4) Correlating average FD with nodal degree and nodal betweenness centrality for each of the 238 nodes (Brainnetome ROIs) using Kendall’s tau procedure with FDR correction to adjust for multiple comparisons. This procedure identified potential associations between motion and the regional network outcome measures.

#### Control analysis only including patients without any treatment sessions before baseline assessment

As baseline measurement was performed around the start of psychotherapy, some patients already had had therapy sessions before the first measurement. As such, seven prospective treatment responders (out of 24) and eight prospective non-responders (out of 22) received (some) treatment before the baseline CAPS interview and fMRI scan (see Table 1). For this reason, it was tested whether observed significant findings were also observed when the analysis focused on only the patients who had not received any treatment before baseline assessment. For head motion, number of motion outliers and IQR of FD were tested as these were significantly different between groups in the original analysis (see “Results” section). For betweenness centrality, the right middle frontal gyrus (MFG), right inferior frontal gyrus (IFG), left inferior temporal gyrus (ITG), right inferior parietal lobule (IPL), right putamen, and left superior parietal lobule (SPL) were tested as these were significantly different between groups in the original analysis (see “Results” section).

#### Exploratory control analysis excluding additional occipital regions for the regional graph analysis

The posterior part of the occipital cortex was not included in the FOV for all participants. As a result, a small amount of occipital ROIs (out of a total of 246, 3.3%) were excluded from graph analyses. We explored the effect of excluding occipital ROIs by testing whether significant findings from the graph analysis were also observed when the analysis removed an extra 8 occipital ROIs around the same location as the already excluded eight occipital ROIs (thus, 16 out of 246 ROIs were excluded); originally excluded eight occipital ROIs: bilateral caudal lingual gyrus, left caudal cuneus gyrus, left middle occipital gyrus, bilateral occipital polar cortex, bilateral inferior occipital gyrus; additionally excluded eight occcipital ROIs: right caudal cuneus gyrus, bilateral rostral lingual gyrus, bilateral ventromedial parietooccpital sulcus, right middle occipital gyrus, and bilateral area V5/MT+. Betweenness centrality in the right MFG, right inferior frontal gyrus (IFG), left inferior temporal gyrus (ITG), right inferior parietal lobule (IPL), right putamen, and left superior parietal lobule (SPL) were tested as these were significantly different between groups in the original analysis (see “Results” section).

## Results

### Participants

Three participants were excluded from fMRI analyses due to excessive scan-to-scan head movement, leading to a sample size of *N* = 46. For the head motion analysis, these participants were included (but excluded at the analysis stage based on statistical outlier detection based on 3*IQR limits, see Supplementary Materials and Methods 1). Twenty-four patients were classified as responders and twenty-two as non-responders. Demographics, clinical data and results of statistical testing for differences between groups are presented in Table 1. The two groups did not differ significantly in demographical or clinical characteristics at baseline. The post-treatment difference in CAPS score was driven by each of the three subscales: re-experiencing, avoiding and hyperarousal. In addition, as expected, post-treatment comorbidity of anxiety disorders was more prevalent in the non-responder group.

### Amplitude of low-frequency fluctuations

No significant differences between the responder and non-responder group were observed for the ROI and exploratory whole-brain analyses.

### Graph analysis

#### Global network analysis

No significant difference between groups was observed for MST connectivity strength (*P* = 0.3638). As a significant correlation with motion was observed for leaf fraction in the motion sensitivity analysis (see paragraph “Sensitivity analyses of head motion potentially confounding graph analysis”), leaf fraction was excluded from analysis of the network integration measures. Omnibus testing revealed no significant main effect of group (*P* = 0.7242), no significant main effect of graph measure (*P* = 1.000), and no significant interaction effect (*P* = 0.2109). Supplementary Figure 2 shows a graphical representation of these findings. These results show that responders exhibited similar levels of global network characteristics compared to non-responders.

#### Regional network analysis

Betweenness centrality in the responder group compared to the non-responder group was (1) significantly lower in the right MFG at the location of the dorsolateral prefrontal cortex (DLPFC; Pcorrected < 0.0119), (2) significantly lower in the right inferior frontal gyrus (rIFG: Pcorrected = 0.0119), (3) significantly lower in the left inferior temporal gyrus (ITL; Pcorrected = 0.0159), (4) significantly higher in the right inferior parietal lobule (IPL; Pcorrected = 0.0286), significantly higher in the right putamen (Pcorrected = 0.0286), and significantly higher in the left superior parietal lobule (SPL; Pcorrected = 0.0317). These results show that the right MFG, right inferior frontal gyrus, and left inferior temporal gyrus were less important hub regions in the responder group compared to the non-responder group, and that the right inferior parietal lobule, right putamen and left superior parietal lobule were more important hub regions. Table 3 provides detailed information and Supplementary Figure 3 provides a graphical representation of the regional betweenness centrality findings.

**TABLE 3:** Test characteristics and associated Brainnetome atlas specifics for regions of interest differing significantly between groups for nodal betweenness centrality.

Region of interest	Hemisphere	*P*-value (FDR corrected)	Median betweenness centrality responders	Median betweenness centrality non-responders	Percentile responders	Percentile non-responders	Brainnetome labels	Brainnetome codes and names	MNI coordinate
Middle frontal gyrus (DLPFC)	Right	<0.0119	0.0210	0.0815	64	94	22	A9/46v, ventral area 9/46	42, 44, 14
Inferior Frontal Gyrus	Right	0.0119	0.0080	0.0170	38	59	30	A44d, dorsal area 44	45, 16, 25
Inferior Temporal Gyrus	Left	0.0159	0.0000	0.0080	11	36	89	A20iv, intermediate ventral area 20	–45, –26, –27
Inferior Parietal Lobule	Right	0.0286	0.0415	0.0080	83	36	140	A40rd, rostrodorsal area 40(PFt)	47, –35, 45
Basal Ganglia (putamen)	Right	0.0286	0.0580	0.0210	90	66	226	vmPu, ventromedial putamen	22, 8, –1
Superior Parietal Lobule	Left	0.0317	0.0290	0.008	75	36	129	A5l, lateral area 5	–33, –47, 50

Percentile indicates the percentile rank of median betweenness centrality for that brain region across all brain regions in the network. *P*-values were corrected for multiple comparisons using false discovery rate (FDR).

### Head motion

Omnibus permutation testing revealed a significant main effect of group (*P* = 0.0011), a significant main effect of motion metric (*P* < 0.00005) and a significant interaction effect (*P* = 0.0009). *Post hoc* testing revealed that the responder group compared to the non-responder group showed (1) a trend for a lower median FD (Pcorrected = 0.0628), (2) a trend for lower maximum FD (Pcorrected = 0.0628), (3) a significantly lower number of motion outliers (Pcorrected = 0.0120), and (4) a significantly lower IQR of FD (Pcorrected = 0.0300). These results show that responders exhibited significantly less head motion than non-responders. Supplementary Figure 4 shows a graphical representation of these findings. No significant correlations were observed between the head motion metrics that differed significantly between groups on one hand and education level, cigarette use at the day of scanning, cigarette use the week prior to scanning, dexterity, age, anxiety and depression on the other hand (all Pcorrected-values > 0.3). Supplementary Table 5 shows test characteristics for these correlations.

### Sensitivity analyses of residual head motion potentially confounding graph analysis

None of the 28203 edges correlated significantly with average FD. The distribution center of the correlations between average FD and edge strength was relatively close to zero (average *r* = –0.0025; see Supplementary Figure 6A). Also, there were no significant correlations between average FD and the global network outcome measures average strength, maximum betweenness centrality, diameter, and mean eccentricity (*r* = –0.056, *P* = 0.583; *r* = 0.184, *P* = 0.072; *r* = –0.092, *P* = 0.377 and *r* = –0.112, *P* = 0.276 respectively). There was, however, a significant correlation between average FD and leaf fraction (*r* = –0.276, *P* = 0.008). Supplementary Figures 6B–F provide a graphical representation of these analyses. In addition, for the regional network analyses, no significant correlations were observed between average FD and nodal degree or nodal betweenness centrality for any of nodes. These results suggest that the preprocessing procedure was relatively effective in mitigating motion effects for the functional connectivity matrix, the regional graph metrics, and four out of the five global graph measures.

### Control analysis only including patients without any treatment session before baseline assessment

As in the original analysis, head motion was significantly lower in the responder group compared to the non-responder group (number of motion outliers: *P* = 0.0061 and interquartile range of framewise displacement: *P* = 0.0333). Also, as in the original analysis, betweenness centrality in the responder group compared to the non-responder group was (1) significantly lower in the right MFG at the location of the DLPFC; Pcorrected < 0.0120, (2) significantly lower in the right IFG: Pcorrected = 0.0032, (3) significantly lower in the left ITL; Pcorrected = 0.0018, (4) significantly higher in the right IPL; Pcorrected = 0.0051, and (5) significantly higher in the left SPL; Pcorrected = 0.0032. No differences were however observed for the right putamen (Pcorrected = 0.3742), which was significantly higher in the responder group in the original analysis. These results show that the analysis is relatively robust to the inclusion of participants who already had had some therapy before the baseline assessment.

### Exploratory control analysis excluding additional occipital regions for the regional graph analysis

As in the original analysis, betweenness centrality in the responder group compared to the non-responder group was (1) significantly lower in the right MFG at the location of the dorsolateral prefrontal cortex (DLPFC; Pcorrected = 0.0008), (2) significantly lower in the right inferior frontal gyrus (rIFG: Pcorrected = 0.0008), (3) significantly lower in the left inferior temporal gyrus (ITL; Pcorrected = 0.0008), (4) significantly higher in the right inferior parietal lobule (IPL; Pcorrected = 0.0014), (5) significantly higher in the right putamen (Pcorrected = 0.0161), and significantly higher in the left superior parietal lobule (SPL; Pcorrected = 0.0200). These results are suggestive that the results of the regional MST analysis may be relatively stable in light of the exclusion of occipital regions.

## Discussion

To our knowledge, this is the first study to investigate amplitude of low-frequency fluctuations (ALFF), MST brain network characteristics, and in-scanner head motion in relation to prospective trauma-focused psychotherapy response in PTSD. We identified that functioning as a hub region in several brain regions and head motion were significantly associated with treatment response. Our findings provide novel insights into neural and behavioral factors predisposing patients with PTSD to benefit from trauma-focused psychotherapy.

### Network analysis

Four out of the six regions with regional differences in network centrality between groups were implicated in a recent meta-analysis of neuroimaging studies assessing PTSD in veterans with combat-related trauma (Boccia et al., 2016). In that study, the right putamen, right IFG, MFG, and IPL were activated relative to controls in studies using paradigms of script-driven imagery, emotional trauma-related stimuli, trauma-related sounds, and pain processing. In the present study, functioning as a hub of a subregion of the right MFG was considerably lower in the responder group, with median betweenness centrality in this group being one-fourth of the non-responder group. This subregion, located in Brodmann areas 9 and 46, is considered to be part of the dorsolateral prefrontal cortex (DLPFC; Cieslik et al., 2013). Interestingly, a recent lesion study observed lateralized functioning of the DLPFC, wherein the left DLPFC is necessary for working memory functioning and the right DLPFC is critical for the manipulation of information in a broad range of reasoning contexts. This was interpreted by the authors that the right DLPFC supports cognitive processes that extend beyond the scope of working memory and enables goal-directed behavior and adaptive decision making (Barbey et al., 2013; Robinson et al., 2014). These are all skills important for effective psychotherapy. Speculating, perhaps the lower functioning as a hub region in responders is related to less involvement of other brain regions in right DLPFC functioning, predisposing these patients for more efficient neural processes in this area, and hence benefiting from psychotherapy. It should be noted that the DLPFC was also implicated in a recent study in which patients with PTSD who exhibited greater recruitment of prefrontal areas (including the right DLPFC) and less recruitment of the amygdala during an emotional reactivity paradigm showed larger improvements in a prospective Prolonged Exposure (PE) psychotherapy intervention. The effects on the left amygdala of transcranial magnetic stimulation (TMS) delivered to the right DLPFC moderated the effect of treatment (Fonzo et al., 2017). These results were interpreted as that the degree of prefrontal control over amygdalar threat detection gates a patient’s capacity to benefit from PE, and a similar mechanism may be at play at the current study, although we observed no differences in the amygdala.

Network centrality of the right IFG was found to be lower in responders compared to non-responders. The right IFG is associated with behavioral inhibition and attentional processes related to the detection of important stimuli (Hampshire et al., 2010; Suda et al., 2020). Its lower importance as a hub in the responder group compared to the non-responder group is less likely to be related to behavioral inhibition, as in an overlapping sample no differences were found between responders and non-responders in behavioral measures of inhibition (Van Rooij et al., 2015). Perhaps the lower network centrality in the responder group is related to its involvement in stimulus detection, making these participants less prone to be distracted by trauma-related and -unrelated cues.

In the present study, the right putamen was more important as a hub region in responders compared to non-responders. Historically, the putamen is known for its involvement in motor functions. However, recently it has become clear that this brain region is implicated in other functions as well, such as the learning of habits (Patterson and Knowlton, 2018), which might provide a link between this region and prospective trauma-focused psychotherapy success. Functioning as a hub of the right IPL was found to be higher in the responder group. The IPL is associated with the mirror neuron system, action initiation, intention, and sense of agency (Fogassi et al., 2005; Rozzi et al., 2008; Tumati et al., 2019). Of note is also a recent study that observed changes in EEG gamma power in this area in the right hemisphere after EMDR (Pagani et al., 2015). Interestingly, in an overlapping sample activity in the IPL during contextual cue processing was predictive of treatment success for PTSD, albeit in the left hemisphere (Van Rooij et al., 2015).

In addition to the four regions observed in the meta-analysis of neuroimaging studies assessing PTSD in veterans described above (Boccia et al., 2016), the left ITG and left SPL were also implicated. The ITG is commonly associated with visual-object processing, and the finding of decreased network centrality in this area in responders might be related to a lower perceptual processing bias for trauma-related stimuli, resulting in decreased triggering of intrusive trauma memories (Kleim et al., 2012). SPL functioning has been related to cognitive reappraisal in patients with mood and anxiety disorders, which might link this region to therapy success in the present study (for a recent meta-analysis see Picó-Pérez et al., 2017). Overall, these findings provide new insights into fMRI resting-state predictors of PTSD psychotherapy treatment success. Previous studies utilizing resting-state fMRI to predict PTSD therapy treatment success observed a diversity of findings, including a combination of within ventral attention network functional connectivity and verbal memory predicting poor psychotherapy treatment response (Etkin et al., 2019), and resting-state networks centered on the superior frontal gyrus and the presupplementary motor area distinguishing treatment responders and non-responders in a sample overlapping with the current study (Zhutovsky et al., 2019). In youth with PTSD, a network within the bilateral STG predicted treatment response, with functional connectivity between the frontoparietal and sensorimotor network being significantly stronger in prospective non-responders than responders (Zhutovsky et al., 2021). Korgaonkar et al. (2020) observed decreased functional connectivity between a variety of networks in prospective treatment responders, while Stout et al. (2021) identified a subgroup of patients associated with improvement in PTSD symptoms from integrated-prolonged exposure therapy. This group showed lower insula to inferior parietal cortex connectivity and higher within cingulate cortex connectivity.

Global network metrics were not different between responders and non-responders. This indicates that the functioning of the entire brain in a network context may not be a sensitive measure to distinguish prospective trauma-focused psychotherapy treatment responders from non-responders, and that regional measures assessing functioning as a hub region can provide additional information.

### Amplitude of low-frequency fluctuations

The absence of differences between responders and non-responders in resting-state brain activity as measured with ALFF suggests that simple, resting-state fMRI fluctuations lack sensitivity to predict PTSD psychotherapy treatment success. A potential explanation is that the relatively complex brain functions involved in cognitive factors predisposing a patient for psychotherapy treatment success are not merely related to differences in resting-state *activity* in specific brain regions, but are more dependent on differences in *functional connectivity*, which is in line with the results of the regional graph analysis. This is in line with several studies showing associations between resting-state measures of functional connectivity and prospective PTSD psychotherapy treatment success (e.g., Etkin et al., 2019; Korgaonkar et al., 2020; Zilcha-Mano et al., 2020). As such, future resting-state studies of prospective PTSD treatment response could focus on measures of functional connectivity and graph analysis.

### Head motion

Two head motion metrics were significantly lower in the responder group compared to the non-responder group. Head motion has a moderate to high level of test-retest stability and is thought to be affected by state and trait components (Couvy-Duchesne et al., 2014; Zeng et al., 2014; Hodgson et al., 2017). The exact mechanism underlying the association between head motion and prospective treatment response is elusive, and future research could investigate this finding further.

### Limitations and methodological considerations

There are several limitations to our study. First, it should be noted that the posterior part of the occipital cortex was not included in the FOV for all participants. As a result, eight occipital ROIs (out of a total of 246, 3.3%) were excluded from the graph analysis, and 11.7% of voxels were excluded from the exploratory whole-brain ALFF analysis. This means that potential differences in ALFF in part of the occipital cortex could not be assessed, and some occipital contributions to the graph analysis could also not be assessed. This may be relevant for the graph analysis, as a previous study observed increased network centrality in the occipital cortex in women victims of sexual assault who later developed PTSD versus trauma-exposed and not-trauma-exposed controls (Quidé et al., 2021). However, the occipital cortex locations reported in that study were included in the current study. In addition, 3.3% is a small amount, and as such impact on the overall structure of the MST is likely to be relatively small. We explored the effect of excluding occipital ROIs by testing if the results of the graph analysis still stood when 8 additional occipital ROIs around the same location as the already excluded occipital ROIs were excluded from analysis. Similar findings were observed, which is suggestive that the results of the graph analysis may be relatively stable in in the light of the exclusion of occipital regions. Still, the findings should be interpreted with this important limitation in mind.

Moreover, the current findings should be interpreted in light of the sample size of the study. Specifically, a power analysis conducted using G*Power software (version 3.1; Erdfelder et al., 1996) revealed that the current sample size could detect a large effect size of *d* = 0.84 with 80% power and an alpha level of 0.05 using a two-tailed independent samples *t*-test. Another limitation is that medication use was only assessed at the fMRI data collection timepoints, and not monitored in between these timepoints. However, no significant differences between groups in any kind of psychoactive medication use were observed at both these timepoints (see Table 1). Also, the patients received a mix of trauma-focused types of psychotherapy. As such, the current findings could not be attributed to a single form of therapy (Zhutovsky et al., 2019). It is also of note that the present study focused on male veterans, and findings may not generalize to civilian and female populations. Finally, patients were included in the study who had already started therapy prior to study participation. This was due to scheduling issues with the scanner, and it was not deemed ethical to suspend treatment for the sake of research. In total, seven prospective treatment responders (out of 24) and eight prospective non-responders (out of 22) received (some) treatment before baseline assessment. However, we do not expect a strong influence on the current results, as a control analysis in which we included only patients who had not received any therapy before baseline assessment led to similar results.

In summary, brain areas involved in executive functioning, attentional processes, learning, action, and visual-object processing exhibited different functioning as a hub in prospective responders to trauma-focused psychotherapy for PTSD compared to non-responders, and head motion was reduced. These results may inform future efforts at individualized treatment selection. Future research should further investigate the mechanistic link between the right DLPFC and trauma-focused psychotherapy treatment success, and explore the mechanism behind head motion as a predictor of PTSD psychotherapy response.

## Data availability statement

Anonymized head motion and preprocessed fMRI data are available upon reasonable request from any qualified investigator. Requests to access the datasets should be directed to RL, R.vanLutterveld@umcutrecht.nl.

## Ethics statement

The studies involving human participants were reviewed and approved by Medical Ethical Committee from Utrecht University. The patients/participants provided their written informed consent to participate in this study.

## Author contributions

RL, SR, MK, and EG: conceptualization. RL, TV, KK, SR, MK, MH, SM, ED, and EG: methodology. RL: formal analysis and writing original draft. SR and MK: data acquisition. RL, TV, KK, SR, MK, MH, SM, ED, and EG: writing – review and editing. All authors contributed to the article and approved the submitted version.
