# Supplementary material for: Spontaneous brain activity, graph metrics, and head motion related to prospective post-traumatic stress disorder trauma-focused therapy response
Source: Front Hum Neurosci. 2022 Aug 12;16:730745. doi: 10.3389/fnhum.2022.730745 (PMC9413840; doi:10.3389/fnhum.2022.730745)
Supplement: Supplementary file 1 [file Data_Sheet_1.docx]

*Supplementary materials and methods S1*

*Global network analysis*

Statistical testing of MST connectivity strength was performed using the ezPerm package with 10,000 permutations in R version 3.6.1 and RStudio Version 1.2.5042 (Lawrence, 2015). Omnibus testing of the network integration measures was performed using the same software (between factor with two levels: responder and non-responder; within factor with four levels: maximum betweenness centrality, leaf fraction, diameter and average eccentricity). Post-hoc testing was performed using the same package in case of statistical significance of the omnibus test, with 10,000 permutations and false discovery rate (FDR) correction for multiple comparisons (Benjamini and Hochberg, 1995). Outliers were identified based on exceeding 3 * IQR limits for connectivity strength and for the same limits for at least 3 of the 4 graph metrics for the omnibus testing, and subsequently removed from analysis. Age and education were included as covariates of no interest for all global network analyses (Guo et al., 2018).

*Regional network analysis*

Differences in degree were tested for each node (i.e., for each ROI) separately, using ezPerm with 10,000 permutations and FDR correction to account for multiple comparisons. Outliers were identified based on exceeding 3 * IQR limits, and subsequently removed from analysis. Age and education were included as covariates of no interest. The same approach was used for betweenness centrality.

*Head motion*

Omnibus testing (between factor with two levels: responder and non-responder; within factor with four levels: median FD, maximum FD, number of motion outliers, IQR of FD) was performed using ezPerm with 10,000 permutations. Outliers were identified based on exceeding 3 * IQR limits for at least 3 of the 4 motion metrics, and subsequently removed from analysis. For motion metrics that differed significantly between groups, correlations were calculated with education level, cigarette use at the day of scanning, cigarette use the week prior to scanning, dexterity, age, anxiety (as measured with the MASQ anxious arousal subscale) and depression (as measured with the MASQ anhedonic depression subscale), with FDR correction for multiple comparisons, and outlier identification and removal based on 3 * IQR limits.

*Supplementary Figure S2*


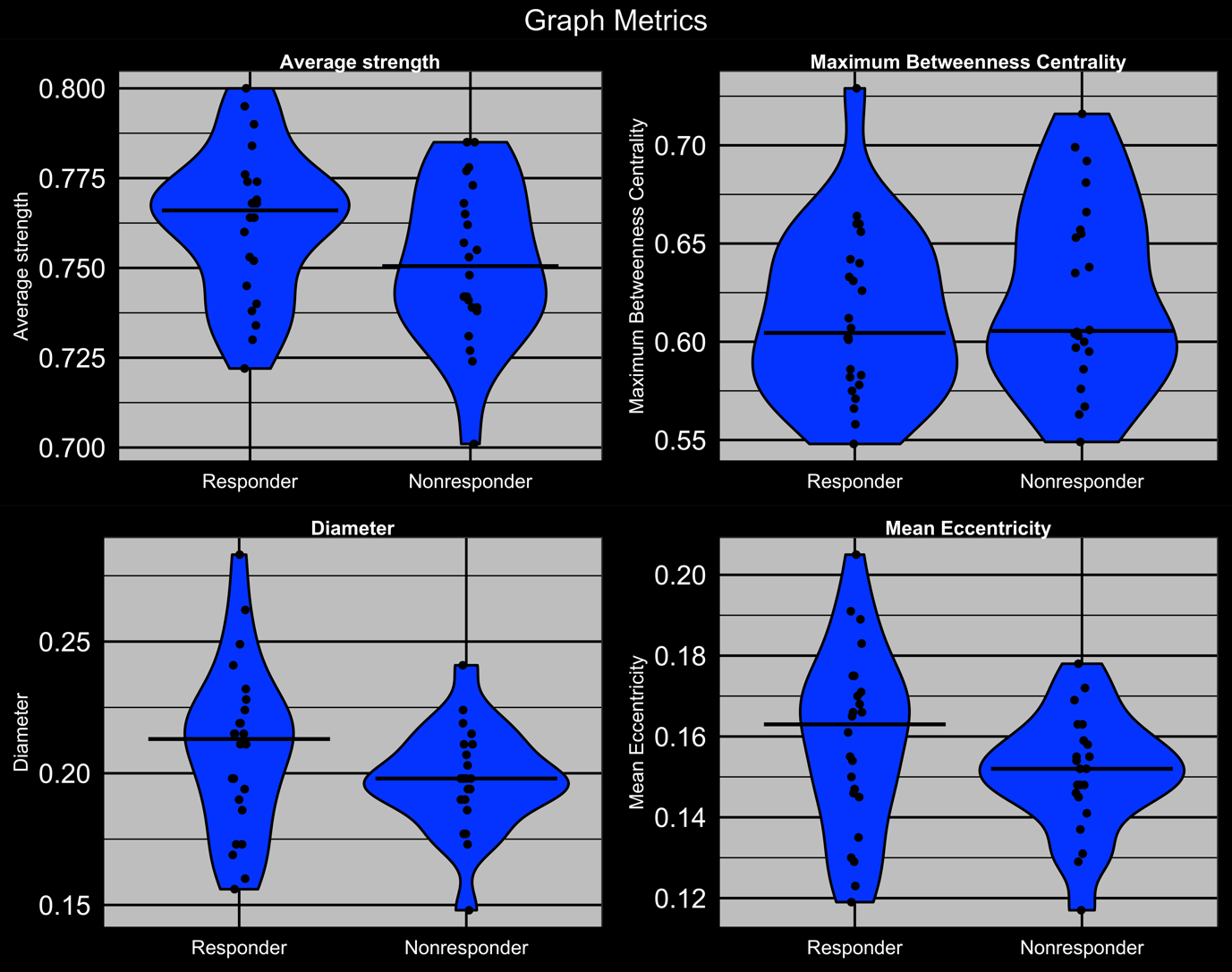


Supplementary Figure S2. Graph metrics. The horizontal bar in each violin plot represents the median. No significant differences were observed between groups.

*Supplementary Figure S3*

*
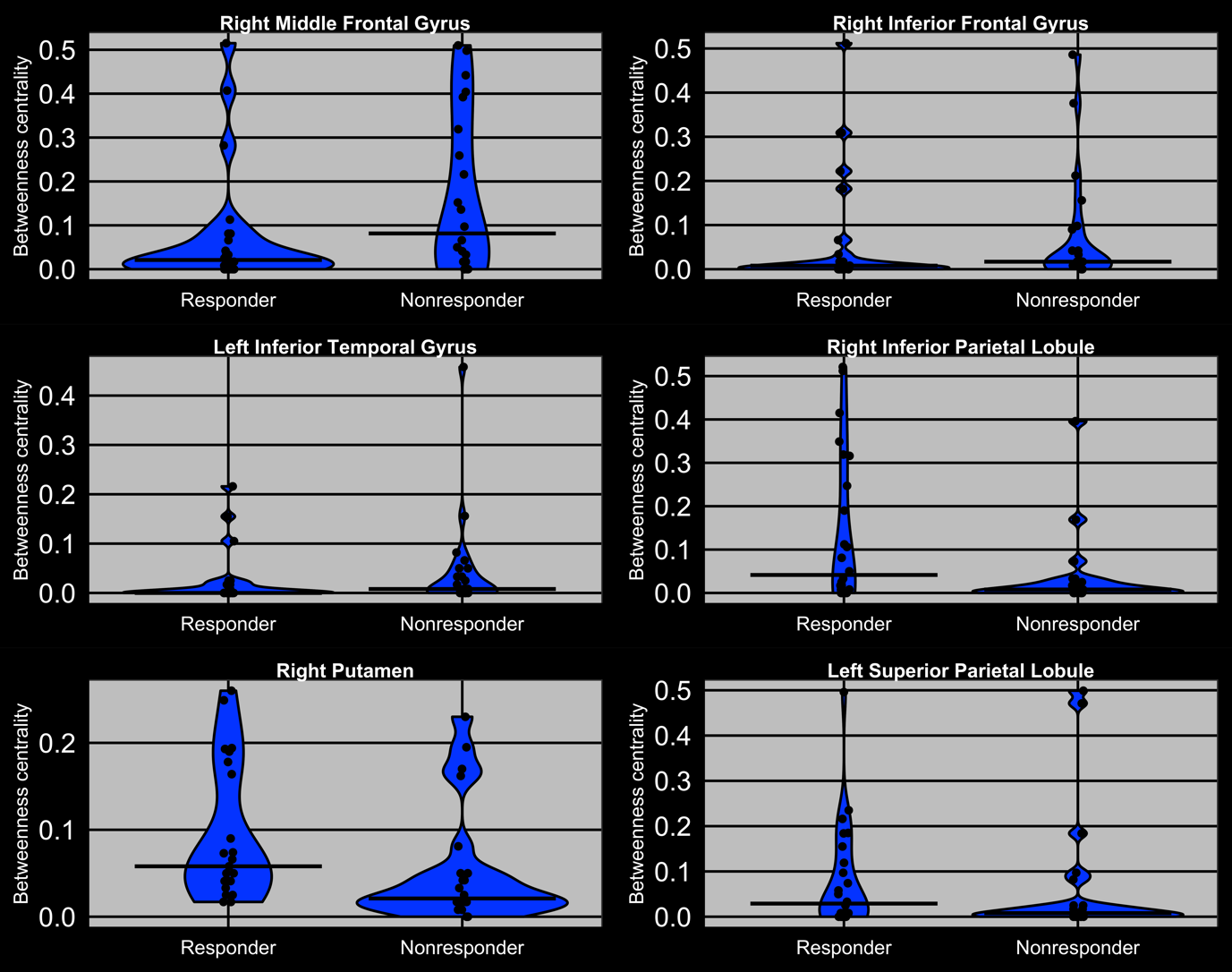
*

Supplementary Figure S3. Regional betweenness centrality for the brain regions showing significant differences (P < 0.05, false discovery rate corrected) between responders and nonresponders. The horizontal bar in each violin plot represents the median.

*Supplementary figure S4*


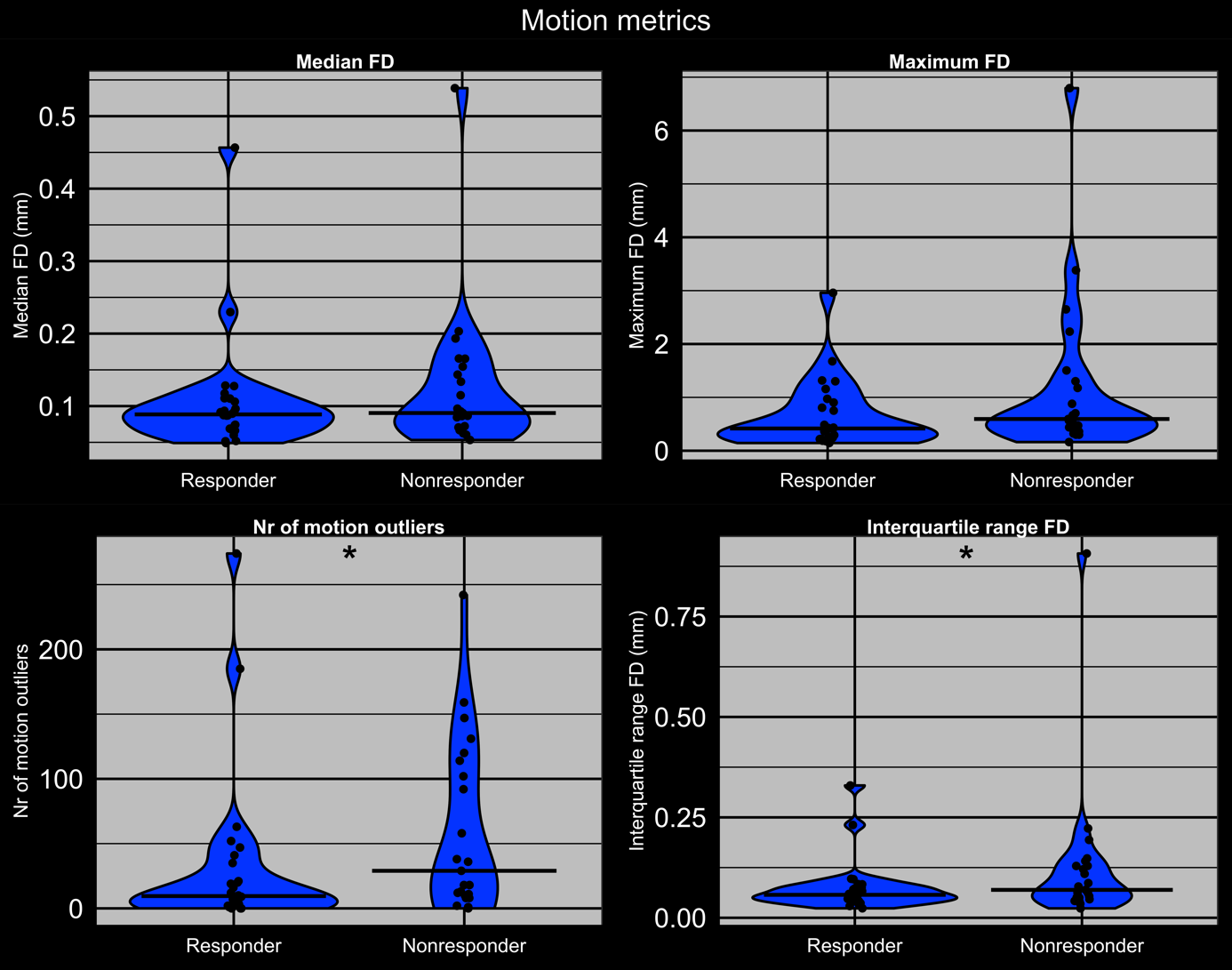


Supplementary figure S4. Motion metrics. The horizontal bar in each violin plot represents the median. FD: Framewise Displacement, *: Statistically significant at P < 0.05 (false discovery rate corrected).

*Supplementary table S5*

Supplementary table S5. Correlation coefficients and associated P-values for motion metrics that differed significantly between groups with individual characteristics. P-values were corrected for multiple comparisons using False Discovery Rate (FDR). FD: Framewise Displacement, IQR: Interquartile Range. Nr of cases with missing data: Cigarette use week prior to scanning: 3; Anxiety: 5; Depression: 5.

| Motion metric | Education level | | Cigarette use day of scanning | | Cigarette use week prior to scanning | | Dexterity | | Age | | Anxiety | | Depression | |
| --- | --- | --- | --- | --- | --- | --- | --- | --- | --- | --- | --- | --- | --- | --- |
|  | r | P | r | P | r | P | r | P | r | P | r | P | r | P |
| Nr of timepoints FD > 0.2 mm | 0.06 | 0.87 | -0.13 | 0.87 | -0.10 | 0.87 | 0.03 | 0.90 | -0.01 | 0.91 | 0.07 | 0.87 | -0.05 | 0.87 |
| IQR of FD | 0.14 | 0.86 | -0.23 | 0.31 | -0.25 | 0.31 | 0.04 | 0.87 | 0.07 | 0.87 | -0.04 | 0.87 | -0.04 | 0.87 |

*Supplementary Figure S6*


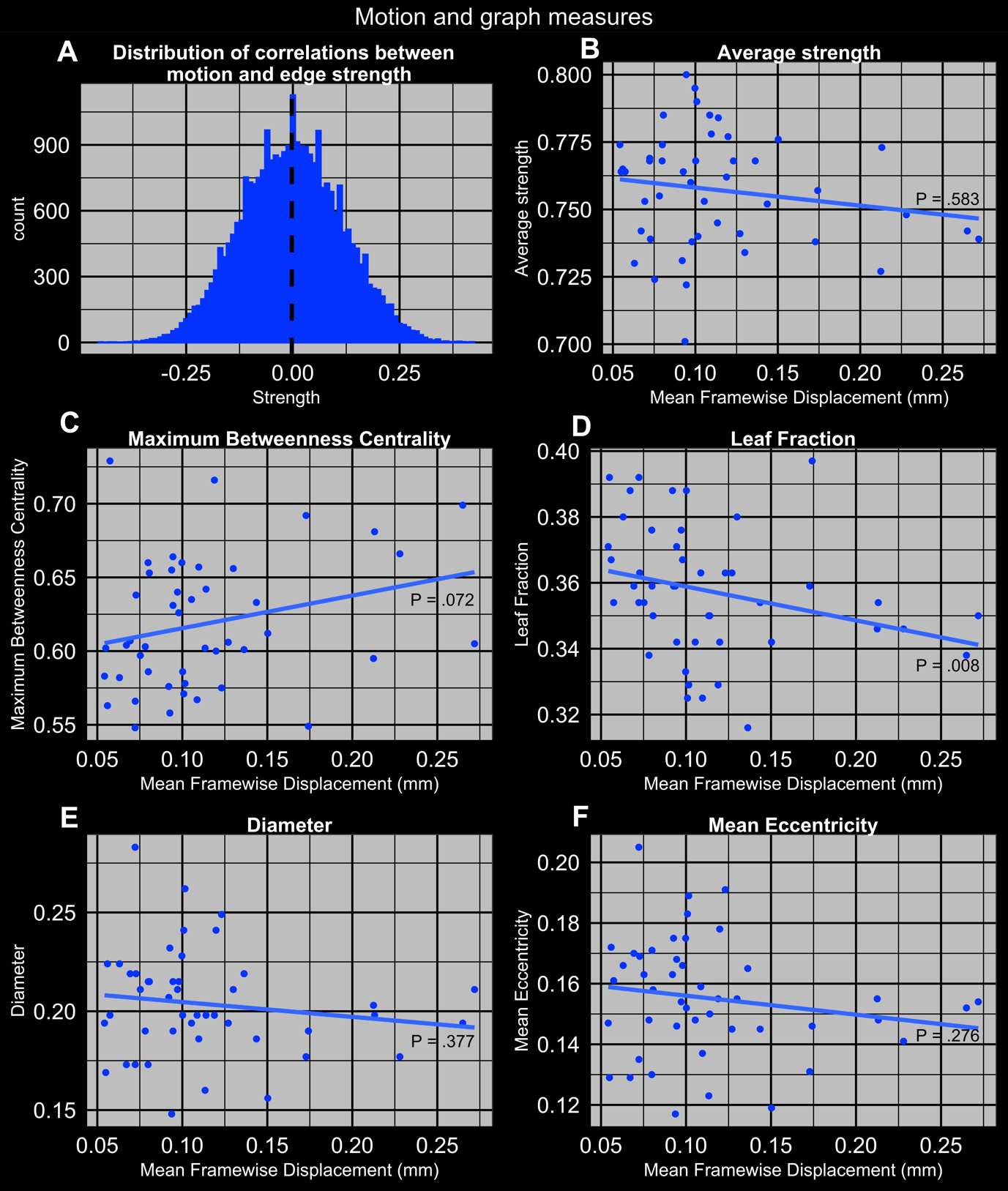


Supplementary Figure S6. (**A**) Distribution of correlations between mean framewise displacement and edge strength. The dashed line indicates the distribution center, which is relatively close to 0 (i.e., 0.0025). (**B - F**) Scatterplots of mean framewise displacement with the five global minimum spanning tree (MST) outcome variables (average strength, maximum betweenness centrality, leaf fraction, diameter and mean eccentricity). Each dot represents a single participant. Average strength, maximum betweenness centrality, diameter and mean eccentricity did not show a significant association with mean motion, while leaf fraction did (see P-values in each graph). P-values were not corrected for multiple comparisons to increase sensitivity of the tests.

*Supplementary references*

Benjamini Y, Hochberg Y (1995) Controlling the false discovery rate: a practical and powerful approach to multiple testing. J R Stat Soc Ser B 57:289–300.

Guo H, Yan P, Cheng C, Li Y, Chen J, Xu Y, Xiang J (2018) fMRI classification method with multiple feature fusion based on minimum spanning tree analysis. Psychiatry Res 277:14–27.

Lawrence MA (2015) ez: Easy Analysis and Visualization of Factorial Experiments. R package (version 4.3). Available at: https://cran.r-project.org/web/packages/ez.
